# Plant‐Pollinator Interactions in Grasslands Established on Arable Land

**DOI:** 10.1002/ece3.73023

**Published:** 2026-02-02

**Authors:** Maria Peer, Sophie Kratschmer, Raja Imran Hussain, Aron Vogel, Matthias Heer, Simon Zwatz, Dietmar Moser, Thomas Frank

**Affiliations:** ^1^ Institute of Zoology, Department of Ecosystem Management, Climate and Biodiversity BOKU University Vienna Austria; ^2^ Department of Botany and Biodiversity Research University of Vienna Vienna Austria

**Keywords:** agroecology, community ecology, insect conservation, mutualistic network, newly established grassland strips, semi‐natural grassland

## Abstract

Newly established grasslands and flower strips on arable land aim to counteract ongoing biodiversity loss, often specifically designed to promote pollinators. However, their effectiveness in supporting diverse and stable plant–pollinator networks remains not fully understood. We compared plant–pollinator interactions in newly established grasslands, 5–6 years after sowing on arable land, and old, permanent grasslands in a Central European agricultural region. Across 1095 recorded interactions, we found significantly higher pollinator visitation frequency and diversity per plant species in newly established grasslands than in old grasslands, particularly for solitary bees and syrphids. Network analyses revealed comparable nestedness and specialization in newly established grasslands and old grasslands. Distinct plant family and color preferences emerged among pollinator groups, with bumble bees favoring Fabaceae, syrphids visiting especially Apiaceae and Rubiaceae, and butterflies preferring Fabaceae and Caryophyllaceae. Several generalist pollinators and key plant species played central roles in both grassland types, highlighting their importance for network connectivity. Our findings suggest that carefully designed new grasslands can support diverse plant–pollinator networks and contribute meaningfully to biodiversity conservation and ecosystem services in agricultural landscapes.

## Graphical abstract

We compared plant‐pollinator networks in newly established and old, permanent grasslands in a Central European agricultural landscape. Newly established grasslands showed higher pollinator visitation frequency and diversity per plant species, especially for solitary bees and syrphids, with a comparable network structure to old grasslands. Our findings suggest that carefully designed new grasslands can support diverse plant‐pollinator networks and contribute to biodiversity conservation in agricultural landscapes.

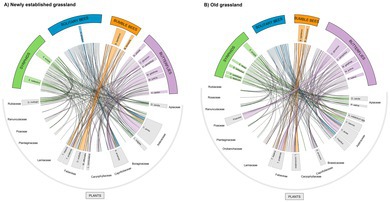

## 1 Introduction

Pollination is fundamental to the reproduction and persistence of plants and considered to be among the most crucial ecosystem services for agricultural production (Klein et al. 2007; Rader et al. 2016). Most animal pollinators of agricultural importance are flower visiting insects, like wild bees, syrphids, butterflies, moths, beetles, or wasps (Dainese et al. 2019). Their pollination service is essential for high yield quality and quantity in crops (Gazzea et al. 2023); pollinator‐dependent crops contribute to 35% of global crop production volume, and more than 75% of all crops are to some extent dependent on pollinators (Klein et al. 2007).

The mutualistic relationship between plants and their pollinators relies on an exchange: plants provide rewards (mainly pollen and/or nectar) for their pollinators, and pollinators guarantee the sexual reproduction of plants as many plants do not self‐pollinate or do so only to a certain degree. Throughout evolution, factors such as competition for resources have shaped the relationship between plants and pollinators, affecting flower traits (color, morphology, odor) and production of pollen and nectar. While many pollinators are generalists and visit a wide range of plant species, others have developed close associations with specific plant species (Nicolson and Wright 2017). In these cases, specialization enables pollinators to use a narrow subset of plants more efficiently (Armbruster 2017). A key feature of robust and biodiversity‐supporting plant‐pollinator networks is a “nested” network structure. This means that specialized species with few links rather share them with generalists than have a different set of links (Bascompte et al. 2003). In plant‐pollinator networks, this nestedness is often asymmetrical: specialized pollinators tend to interact with generalist plant species, and vice versa. The generalist species tend to have more stable population abundances, making them reliable interaction partners for specialist species (Bascompte et al. 2006). Nested and asymmetrical properties of pollination networks reduce interspecific competition and enhance the number of coexisting species; in addition, they make them more tolerant to species loss associated with habitat transformation and disturbance (Bastolla et al. 2009; Thébault and Fontaine 2010). Beyond that, high numbers of different pollinator species, as well as high numbers of interactions, increase the chances of pollination at any given time and location (Borchardt et al. 2021; Garibaldi et al. 2014).

Nevertheless, even the loss of single species may have negative impacts on plant‐pollinator networks (Brosi et al. 2017), which is worrying in times of pollinator declines due to habitat loss, pesticides, invasive alien species and climate change (Potts 2016; Potts et al. 2010). The loss of ecological interactions is an important component of biodiversity loss and may frequently precede species extinctions and therefore needs to be considered when assessing ecosystems (Valiente‐Banuet et al. 2015).

An approach that has been increasingly gaining attention in recent years is network modeling (Borchardt et al. 2021). Species interaction networks offer insights into the complexity of plant‐pollinator interactions by illustrating webs of relationships among species and analyzing all documented “links” between interacting species (e.g., plants and pollinators) and how their distributions are spread (Bascompte 2009). Embracing a systems‐ecology approach shifts the focus from individual species to interactions within the broader ecosystem and is the most promising way to simultaneously improve the conservation efforts for pollinators, including newly established grasslands, and the sustainability of agriculture (Borchardt et al. 2021).

Newly established grasslands in agricultural landscapes aim to counteract biodiversity loss—and are often specifically designed to promote pollinators. Recent studies found great benefits of grassland strips for pollinators, especially if they showed a high number of plant species and high flower abundance (Albrecht et al. 2007; Hussain et al. 2023; Peer et al. 2024). However, while most studies concentrate on taxonomic richness within grassland strips, the study of plant‐pollinator networks in newly established grassland has only gained attention in recent years (Hadrava et al. 2022). Studies in France and the Czech Republic found more diverse plant‐pollinator networks in permanent grassland than in newly established grassland (Hadrava et al. 2022; Michelot‐Antalik et al. 2021). However, the effectiveness of newly established grassland on arable land in supporting diverse and stable plant–pollinator networks remains not fully understood, particularly regarding differences among taxonomic pollinator groups, such as butterflies, bees, or syrphids.

The aim of this study is to provide a detailed analysis of plant visitation patterns in a community of pollinators in Central European grasslands and to find out how the plant visitation network differs among four pollinator insect groups: solitary bees, bumble bees, syrphids and butterflies. We explored the structural features of the plant‐pollinator network and wanted to find out if visitation frequency, visitation diversity, specialization, nestedness, and connectance differ between old, permanent (“OG”) versus newly established grasslands (“NG”). These network metrics provide complementary insights into community structure: visitation frequency and diversity reflect pollinator activity and resource use, specialization indicates the degree of reliance on specific partners, nestedness describes the hierarchical structure of interactions, and connectance measures the proportion of realized interactions (Dunne et al. 2002; Blüthgen et al. 2006). Further, we wanted to study the diet overlap of solitary bees, bumble bees, syrphids and butterflies in the different grassland types. Finally, we also tested whether plant flower color and plant family affect plant visitation rates by pollinators from different taxa. We studied plant‐pollinator networks in newly established grasslands and old grasslands in 2021 and 2022—five to six years post‐establishment of newly established grasslands.

Based on earlier research that showed significantly higher wild bee abundance and species richness in newly established grasslands compared to old grasslands (Peer et al. 2024), and because of the assumption that greater pollinator abundance leads to increased plant interaction frequency and diversity (Burkle and Alarcón 2011), we hypothesize that visitation frequency and diversity of pollinators will be higher in newly established grasslands than in old grasslands. In contrast, syrphids and butterflies showed similar abundance and species richness in both grassland types, suggesting similar visitation patterns. Although pollinator assemblages did not differ significantly between newly established grassland and old grassland in the studied years (Peer et al. 2024), fewer feeding specialists among wild bees were observed in newly established grassland (Peer et al. 2025), which—given that specialists tend to have fewer plant partners and thus influence network structure (Blüthgen et al. 2006)—leads us to expect differences in network structure. Moreover, because plant assemblages differed between grassland types (Peer et al. 2024), we expect the composition of visited plant species to be different.

## 2 Methods

**FIGURE 1:**
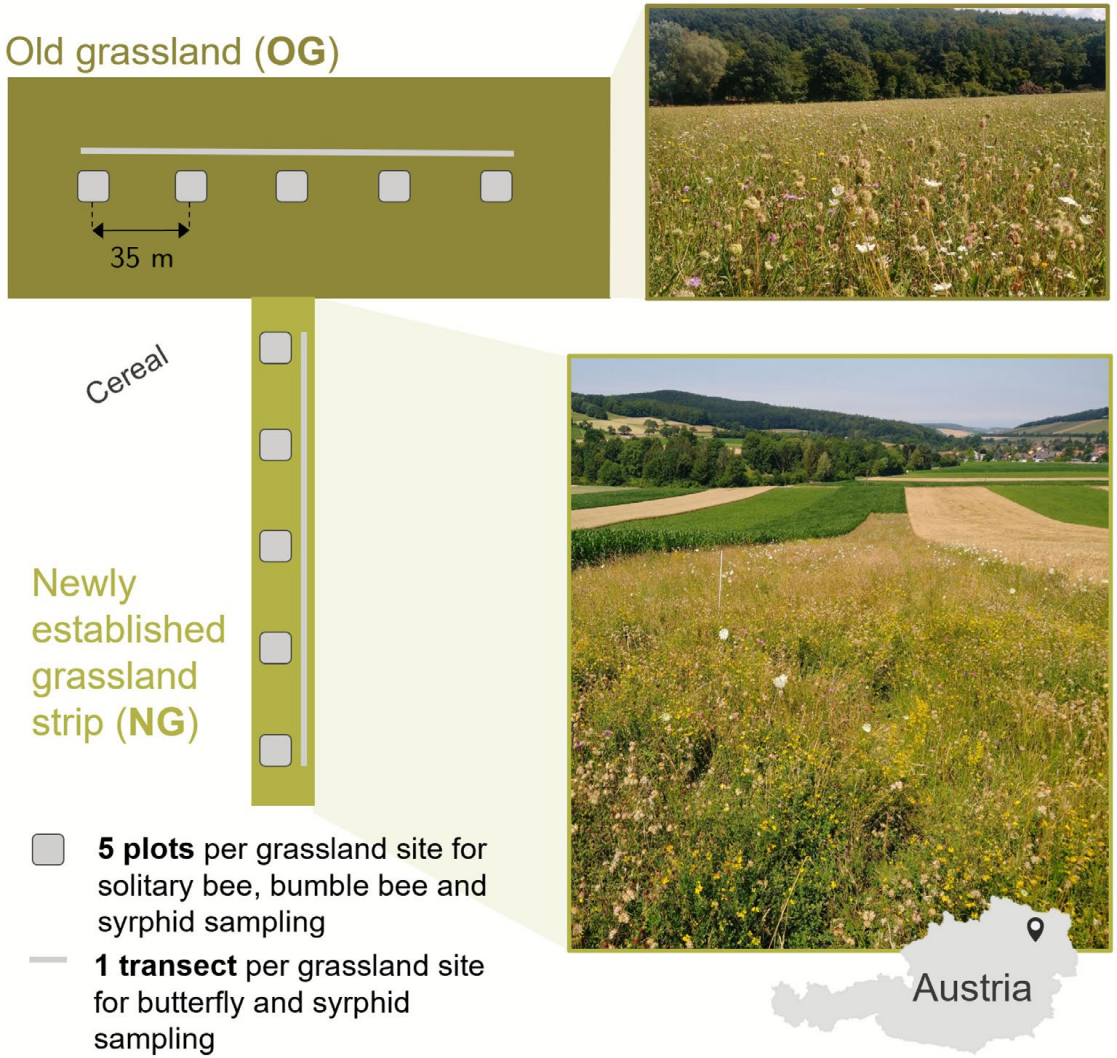
Study design. Solitary bee, bumble bee, syrphid, and butterfly interactions with plants were recorded in five replicates (= grassland sites) of newly established grassland and old grassland in the fifth (2021) and sixth (2022) year after establishment of newly established grassland. A map and overview of the locations of the study sites is given in the (Appendix A1).

Sampling was conducted in Sieghartskirchen, which is located in the intensively managed agricultural region “Tullnerfeld” in Lower Austria (48°16′02.5″ N 16°05′07.9″ E, 48°15′08.3″ N 16°02′56.9″ E). The mean annual air temperature of this region is 9.9°C, and the mean annual precipitation is 673 mm (ZAMG, 2021). We sampled flower visitors in two types of extensively managed grassland—newly established grassland strips and old, permanent grasslands—with five replicates per grassland type (Figure 1):

- **Newly established grasslands** were sown in 2016 on former arable land with a flower‐ and species‐rich plant composition of 41 characteristic native plant species that mimic the plant assemblage of old grassland in the region (see Brandl et al. 2022 for plant species used for the seed mixture). All sites were 10 m wide and study plots were distributed along 175 m long transects. Newly established grasslands were mown once a year earliest on 1st July and the biomass was removed. They were surrounded by cereal fields and the short side bordered on old grasslands. In the studied years (5 to 6 years post‐establishment) newly established grasslands showed on average (±SD) 38 ± 3 plant species and a flower frequency of 30% ± 28% per site (see Peer et al. 2024 for flower frequency sampling method).
- **Old grasslands** are permanent, long‐existing semi‐natural grasslands, on average 2 ha in size, which are typical meadows for hay production and get cut two times per year. In the study period, old grasslands hosted on average (±SD) 43 ± 13 plant species and flower frequency was 34% ± 24% per site.

Four different pollinator groups—butterflies (Rhopalocera), syrphids (Syrphidae), bumble bees (*Bombus* spp.) and solitary bees (Anthophila)—were recorded. Plant visitations in old grassland and newly established grassland were sampled four times per year in a monthly interval between May and August in 2021 (solitary bees, bumble bees, butterflies) and 2022 (solitary bees, bumble bees, butterflies, syrphids). We recorded all interactions of pollinators with plants. This mainly comprised floral visits, but in the case of syrphids and butterflies, it also included non‐floral visits (e.g., honeydew feeding, egg‐laying on leaves). Butterfly‐plant interactions were recorded by 20 min transect walks along the entire grassland, with the observer walking in zigzags and documenting interactions within a 5 m radius in front. Interactions of solitary bees, bumble bees and syrphids with plants were recorded in five plots (= fixed observational units), arranged in 35 m distances along a transect at each site. Solitary bees and syrphids were recorded in 2 m^2^ plots and bumble bees were recorded in 10 m^2^ plots, for 10 min in each plot. Additionally, syrphid‐plant interactions were also recorded by 20 min transect walks. Sampling methods and spatial scales differed among pollinator groups to account for variation in detectability and mobility of different species. Further details on sampling methods are provided in Peer et al. (2024).

Sampling was conducted on days with a minimum of 15°C air temperature and low wind speed (< 4 Beaufort). Individuals of bees and syrphids that could not be determined in the field to species level were identified in the laboratory, and butterflies were exclusively identified in the field to species level. Each grassland type had five replicates. In addition to the sampling of plant‐pollinator interactions, plant abundance of herbs, grasses, and legumes was measured in newly established grassland and old grassland once per year using the Braun‐Blanquet scale (Dengler et al. 2008), and following Fischer et al. (2008) (Mean cover of plant species in newly established grassland and old grassland in Appendix A6).

Flower colors of each plant species were recorded in the field and compared with entries in the Plant Trait Database (Kattge et al. 2020).

## 3 Statistical Analysis

To visualize plant‐pollinator networks we created chord diagrams using the package circlize (Gu et al. 2014).

To compare the structural features of the plant‐pollinator networks of different grassland types (newly established grassland and old grassland) and of different pollinator groups (solitary bees, bumble bees, syrphids and butterflies) we calculated several network metrics. At the plant‐species level, we assessed the number of pollinator species visiting recorded plant species at each grassland type (visitation diversity) and calculated visitation frequency as the total number of pollinator visits per plant species. These metrics reflect the overall pollination potential and the potential redundancy of pollination services. We tested for differences in visitation frequency and diversity between newly established grassland and old grassland using a generalized linear mixed‐effects model (GLMM) fitted with a Poisson distribution with random intercepts for sites and plant species. We used the residual diagnostics available in the DHARMa package to assess dispersion, zero‐inflation and homogeneity of variance (Hartig 2022). In the case of zero‐inflated data, we used the function glmmTMB() to build generalized Poisson or binomial family GLMMs (package glmmTMB, Brooks et al. 2017).

Further, we calculated the following bipartite network indices using the function “networklevel” in the R package bipartite (Dormann et al. 2009):

- Connectance: measures the fraction of all possible links that are realized in a network (= sum of links divided by number of cells in the matrix) (Dunne et al. 2002)
- Network‐wide specialization (H2′): H2′ is an index that quantifies the level of “complementarity specialization” or the degree of niche partitioning across an entire bipartite network. It measures the extent to which observed interactions differ from those expected based on species’ total number of interactions (Blüthgen et al. 2006). It ranges between 0 (no specialization) and 1 (complete specialization).
- Nestedness: occurs when less‐connected species tend to interact with subsets of the species that more‐connected species interact with, forming a hierarchical pattern of interactions. The NODF index (“Nestedness based on Overlap and Decreasing Fill”) quantifies nestedness by evaluating how much the interactions of less‐connected species overlap with, and are subsets of, those of more‐connected species. NODF ranges from 0 (not nested) to 100 (perfectly nested) (Almeida‐Neto et al. 2008).

To compare network indices of networks with different size and species abundance patterns, we standardized network indices to z values by comparing them to expectations from a null model: $z-\text{value}=\frac{\text{observed metric}-\text{mean metric of null model}}{\text{standard deviation of null model}}$. Null models were created with the “nullmodel” function in the package bipartite (1000 random permutations per index). Computation of network indices and null model analysis was based on the description of Dormann (2024). To control for potential bias due to differences in sampling intensity, we rarefied the number of interactions to a fixed maximum possible count (*n* = 100 for grassland type comparison, *n* = 40 for pollinator group comparisons) and recalculated network indices (e.g., H2′) across random draws (*n* = 100) and compared them to our results (Appendix A5). This approach follows established procedures in network ecology (Dormann et al. 2009).

Differences in diet niches among pollinator groups were tested with a PERMANOVA (Bray‐Curtis dissimilarities, 999 permutations) using the adonis function from the vegan package (Oksanen et al. 2022). To make pairwise comparisons between pollinator groups, an adonis test using the pairwise.adonis() function from the pairwiseAdonis package was performed (Martinez Arbizu 2020). To visualize diet niche overlaps, we did non‐metric Multidimensional Scaling (NMDS).

Finally, we also tested whether plant flower color and plant family affect flower visitation rates by pollinator groups conducting a fourth‐corner analysis. Observations were aggregated in a matrix (S) indicating the frequency of pollinator species (rows) visiting plant species (columns). Correspondingly, a matrix (E) giving the pollinator group for each species and a trait matrix (T) giving the traits for each plant species were created. Fourth corner analysis fits a predictive model for the abundance of pollinators visiting different plants (S), as a function of E, T and E–T interactions (Brown et al. 2014). We fitted models using the function traitglm() of the package mvabund (Wang et al. 2022). To account for different sampling methods for pollinator groups, we used the composition = TRUE option in the traitglm() function, which models relative abundances. A Lasso penalty was applied to reduce the environmental–trait relationships to 0, should they not improve the fit of the model. This means that non‐zero estimates should be treated as significant.

We analyzed data with R version 4.3.3 in RStudio 2024.12.0 (Posit team 2024; R Core Team 2024).

## 4 Results

**FIGURE 2:**
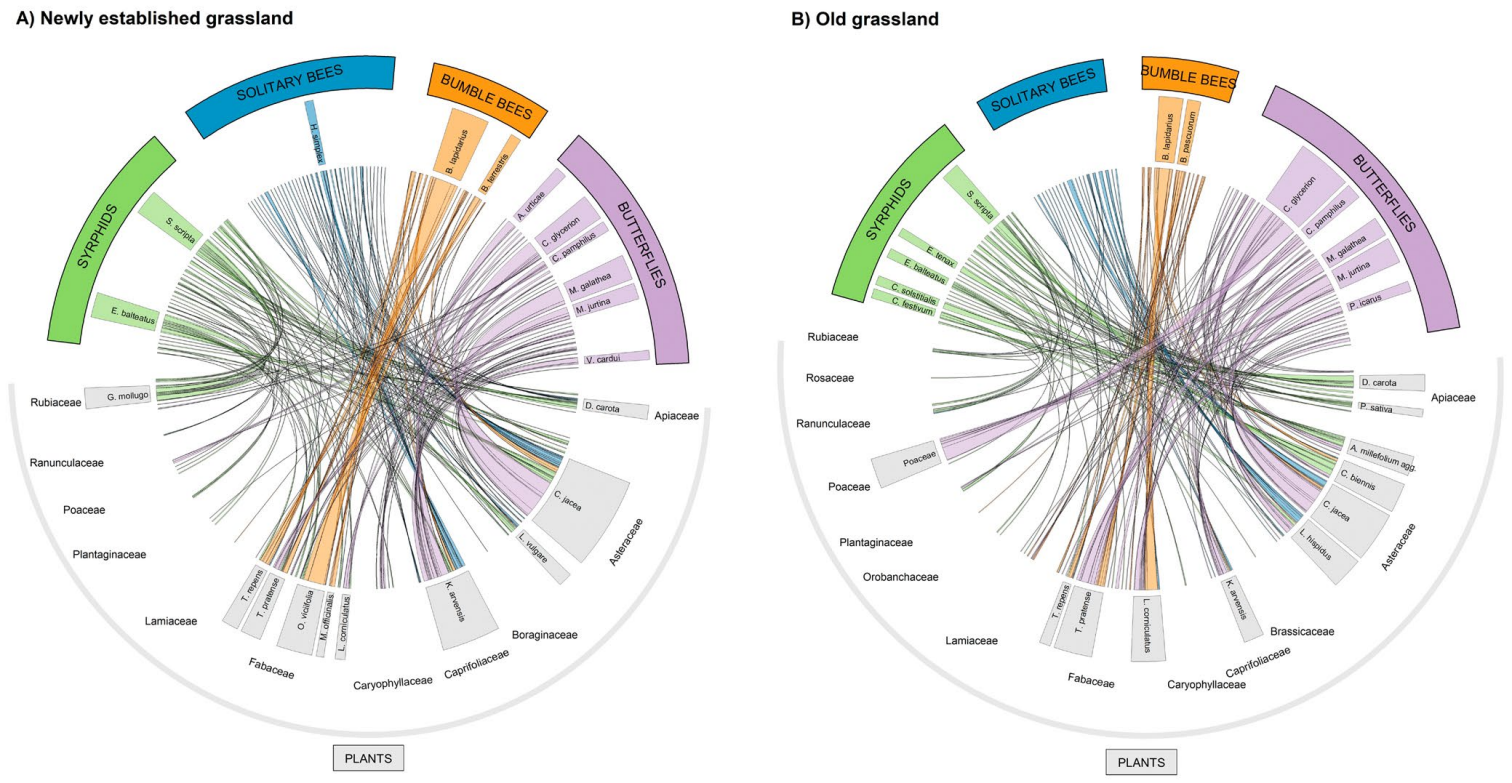
Chord diagrams of plant‐pollinator interactions in (A) newly established grassland and (B) old, permanent grassland. Interactions between pollinator groups (syrphids, solitary bees, bumble bees, butterflies) and plant families (e.g., Apiaceae, Asteraceae) are shown. Lines connecting the two halves represent interactions, with line thickness reflecting the number of interactions. Full species names: Syrphids: *Episyrphus balteatus*, *Sphaerophoria scripta* , *Eristalis tenax* , *Chrysogaster solstitialis*, *Chrysotoxum festivum*; Solitary bees: *Halictus simplex* agg.; Bumble bees: *Bombus lapidarius* , *Bombus pascuorum* , *Bombus terrestris* agg.; Butterflies: *Aglais urticae* , *Coenonympha glycerion*, *Coenonympha pamphilus*, *Melanargia galathea*, *Maniola jurtina*, *Polyommatus icarus*, *Vanessa cardui*; Plants: *Galium mollugo* , *Trifolium repens* , *Trifolium pratense* , *Onobrychis viciifolia* , *Melilotus officinalis* , *Lotus corniculatus* , *Knautia arvensis* , *Leucanthemum vulgare* , *Leontodon hispidus* , *Centaurea jacea* , *Crepis biennis* , *Achillea millefolium* , *Pastinaca sativa* , *Daucus carota*.

We recorded 1095 plant‐pollinator interactions in newly established grasslands and old grasslands, including 135 solitary bee individuals (43 species) visiting 18 plant species from 9 families, 204 bumble bee—plant interactions (11 bee species, 18 plant species from 6 families), 301 syrphids from 28 different species visiting 37 plant species (11 families), and 455 butterfly—plant interactions (30 butterfly species, 33 plant species from 10 families). All interactions are visualized in chord diagrams (Figure 2).

**FIGURE 3:**
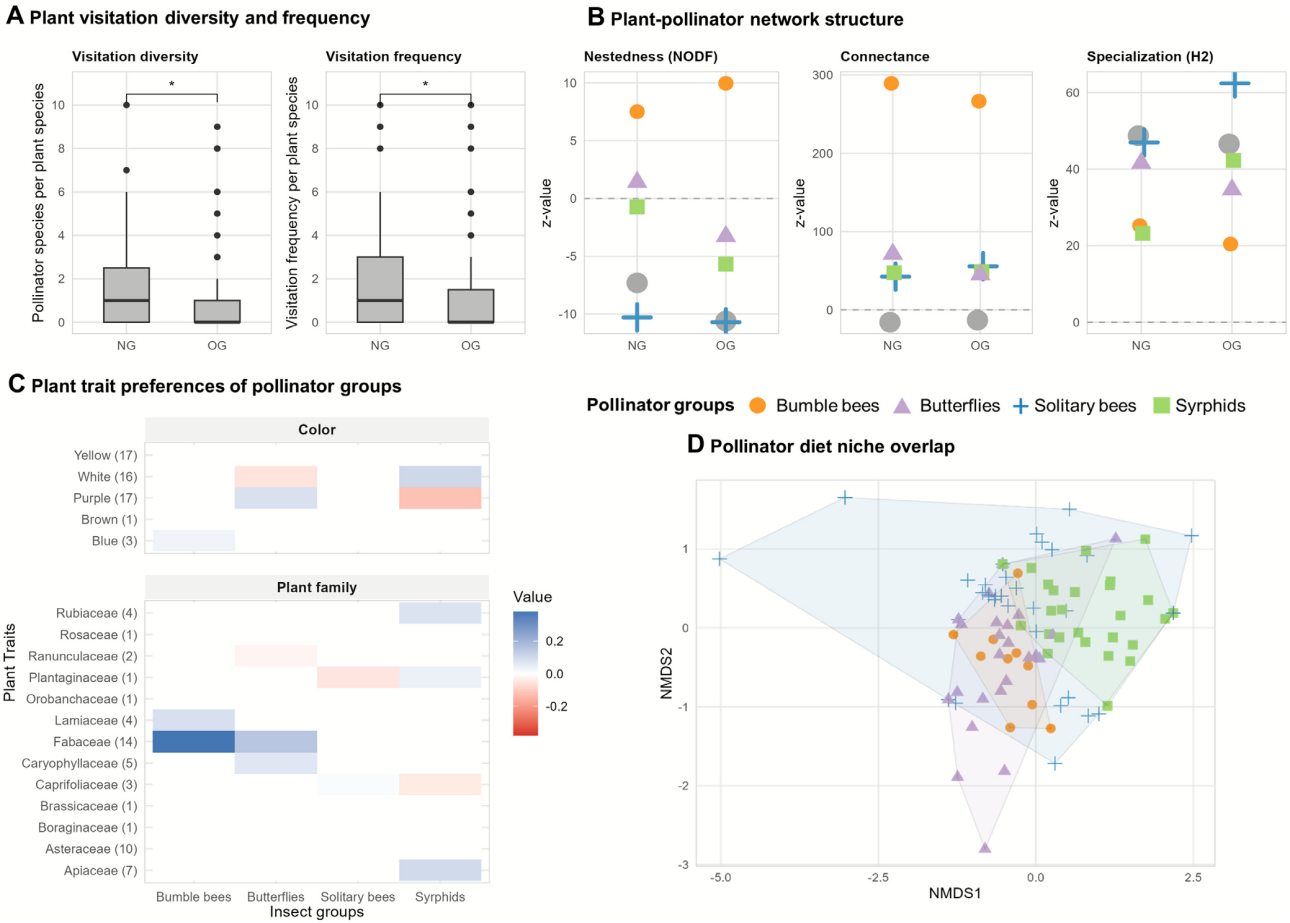
(A) Plant visitation diversity and frequency in NG (“newly established grassland”) and OG (“old grassland”). Boxplots show significantly higher visitation diversity and frequency in NG compared to OG (*p* < 0.05, glmer), outliers above *y* = 10 are not shown. (B) Metrics of plant‐pollinator network structure in NG (“newly established grassland”) and OG (“old grassland”). Z‐values of connectance, nestedness and specialization express the difference of the full network (gray dots) and subsets (different pollinator groups) to the null model (dashed line). (C) Fourth‐corner modeling results for bumble bee, butterfly, syrphid and solitary bee plant visitation. Colors represent the strength of fourth‐corner coefficients (shading) and their direction (blue = significant positive, red = significant negative). Numbers in brackets indicate the number of plant species with the respective trait. Note that some traits are unique to single plant species. (D) Nonmetric multidimensional scaling (NMDS) of diet niches (plant preferences) of pollinator groups bumble bees, butterflies, syrphids and solitary bees. Pollinator groups overall had a significant effect on diet niches (PERMANOVA, *F* = 1.5349, *p* < 0.05). Significant pairwise differences were detected between diet niches of butterflies and syrphids (PERMANOVA, *F* = 2.22, *p* < 0.05).

Pollinator visitation frequency and diversity per plant species were significantly higher in newly established grasslands compared to old grasslands (Figure 3A). This pattern was especially pronounced for solitary bees and syrphids (Appendices A2 and A3). In all models, plant species identity (as a random factor) accounted for a large proportion of the variance, while variation among sites was low or negligible (Appendices A2 and A3). In both grassland types, most pollinator visits (21% in total) were recorded on *Centaurea jacea*, and the 10 most visited plant species belonged to the families Asteraceae, Fabaceae, Caprifoliaceae, and Apiaceae; in old grassland, pollinators (especially butterflies) visited Poaceae more frequently than in newly established grassland (Table 1, Figure 2). Plants that were frequently visited in newly established grassland but not in old grassland were, for example, *Galium mollugo* by syrphids or *Knautia arvensis* by solitary bees (Figure 2).

**TABLE 1:** Top ten most frequently visited plant species in newly established grassland and old grassland.

	Newly established grassland				Old grassland			
	Species name	Plant family	Pollinator abundance	Pollinator species richness	Species name	Plant family	Pollinator abundance	Pollinator species richness
1	*Centaurea jacea*	Asteraceae	171	38	*Centaurea jacea*	Asteraceae	65	19
2	*Knautia arvensis*	Caprifoliaceae	108	36	*Trifolium pratense*	Fabaceae	49	15
3	*Onobrychis viciifolia*	Fabaceae	66	12	*Lotus corniculatus*	Fabaceae	44	16
4	*Galium mollugo*	Rubiaceae	38	14	*Leontodon hispidus*	Asteraceae	41	18
5	*Trifolium pratense*	Fabaceae	38	15	*n.i*.	Poaceae	39	8
6	*Trifolium repens*	Fabaceae	31	10	*Crepis biennis*	Asteraceae	38	14
7	*Daucus carota*	Apiaceae	26	16	*Knautia arvensis*	Caprifoliaceae	26	14
8	*Leucanthemum vulgare*	Asteraceae	26	15	*Achillea millefolium*	Asteraceae	25	13
9	*Lotus corniculatus*	Fabaceae	18	6	*Daucus carota*	Apiaceae	21	9
10	*Melilotus officinalis*	Fabaceae	14	4	*Trifolium repens*	Fabaceae	14	8

*Note:* “n.i., Poaceae” (old grassland) represents aggregated data for multiple unidentified species.

Pollinator species that visited the highest numbers of different plant species were *Coenonympha glycerion*, *Sphaerophoria scripta* , *Episyrphus balteautus*, *Maniola jurtina*, and *Bombus pascuorum* (Table 2). Of the 43 (old grassland) and 38 (newly established grassland) available plant species, in each grassland type, 15 plant species covered the total pollinator species community observed (Table 3). Six plant species from the families Fabaceae, Asteraceae, Caprifoliaceae, and Apiaceae were visited by more than 75% of observed pollinator species (taxa) in the respective grassland type (Table 3).

**TABLE 2:** Top five pollinator species in newly established grassland and old grassland visiting the highest number of plant species.

	Newly established grassland				Old grassland			
	Species name	Pollinator group	Plant visits	Plant species number	Species name	Pollinator group	Plant visits	Plant species number
1	*Sphaerophoria scripta*	Syrphids	47	17	*Coenonympha glycerion*	Butterflies	71	14
2	*Episyrphus balteatus*	Syrphids	46	14	*Sphaerophoria scripta*	Syrphids	24	11
3	*Coenonympha glycerion*	Butterflies	49	9	*Maniola jurtina*	Butterflies	33	9
4	*Bombus lapidarius*	Bumble bees	70	7	*Bombus pascuorum*	Bumble bees	17	9
5	*Bombus pascuorum*	Bumble bees	13	7	*Coenonympha pamphilus*	Butterflies	20	8

**TABLE 3:** Minimum number of plant species that cover the total pollinator community in newly established grassland and old grassland. Plant species are listed in order of their cumulative contribution to the total pollinator species richness (% accumulated).

	Newly established grassland			Old grassland		
	Plant species	Plant family	Covered pollinator species (accumulated %)	Plant species	Plant family	Covered pollinator species (accumulated %)
1	*Centaurea jacea*	Asteraceae	42%	*Centaurea jacea*	Asteraceae	26%
2	*Knautia arvensis*	Caprifoliaceae	58%	*Lotus corniculatus*	Fabaceae	44%
3	*Daucus carota*	Apiaceae	73%	*Crepis biennis*	Asteraceae	58%
4	*Leucanthemum vulgare*	Asteraceae	80%	*Daucus carota*	Apiaceae	66%
5	*Trifolium pratense*	Fabaceae	84%	*Leontodon hispidus*	Asteraceae	73%
6	*Onobrychis viciifolia*	Fabaceae	86%	*Knautia arvensis*	Caprifoliaceae	78%
7	*Knautia longifolia*	Caprifoliaceae	88%	*Pastinaca sativa*	Apiaceae	84%
8	*Lotus corniculatus*	Fabaceae	90%	*Trifolium repens*	Fabaceae	88%
9	*Galium mollugo*	Rubiaceae	92%	*Ranunculus acris*	Ranunculaceae	90%
10	*Silene dioica*	Caryophyllaceae	93%	*Trifolium pratense*	Fabaceae	92%
11	*Myosotis* sp.	Boraginaceae	95%	*Vicia sativa*	Fabaceae	93%
12	*Trifolium repens*	Fabaceae	96%	*Ajuga reptans*	Lamiaceae	95%
13	*Trifolium hybridum*	Fabaceae	97%	*Plantago lanceolata*	Plantaginaceae	96%
14	*Dianthus carthusianorum*	Caryophyllaceae	98%	*Anthoxanthum odoratum*	Poaceae	97%
15	*Leontodon hispidus*	Asteraceae	99**%**	*Taraxacum officinale agg*.	Asteraceae	99%
16	*Medicago lupulina*	Fabaceae	100%	*Ranunculus bulbosus*	Ranunculaceae	100%

Network structure varied only slightly between grassland types. Newly established grassland networks exhibited slightly higher nestedness (NODF = 24.4) and specialization (H2′ = 0.42) than old grassland (NODF = 18.3, H2′ = 0.41; Figure 3B). Connectance, which measures interaction density, was higher than the null model across all pollinator groups, with bumble bees showing the highest connectance (old grassland: 0.35, newly established grassland: 0.38). Nestedness (NODF) was higher than the null model for bumble bees and butterflies in newly established grassland, while other pollinator groups showed lower metrics than the null model. Specialization (H2′) was highest for solitary bees (H2′_OG_ = 0.5) and lowest for bumble bees (H2′_OG_ = 0.3), particularly in old grassland (Figure 3B).

Overall, Asteraceae and Fabaceae received the highest number of visits across all pollinator groups (Figure 2). However, when comparing pollinator groups, they displayed clear differences in their plant family preferences (Figure 3C). Bumble bees showed a strong preference for Fabaceae and also favored Lamiaceae, butterflies preferred Fabaceae and Caryophyllaceae, syrphids were associated with Rubiaceae and Apiaceae, and solitary bees with Caprifoliaceae (Figure 3C). Bumble bee visits to Fabaceae were mostly to *Onobrychis viciifolia* in newly established grassland and *Lotus corniculatus* in old grassland (Figure 2). Syrphids were most often observed on *Daucus carota* in old grassland and newly established grassland and *Galium mollugo* in newly established grassland (Figure 2). Color also influenced visitation choices of some pollinator groups according to the fourth corner results: butterflies preferred purple and syrphids favored white flowers (Figure 3C).

Diet niches were significantly different among pollinator groups (Figure 3D, PERMANOVA, *F* = 1.5349, *p* < 0.05). Pairwise comparisons showed significant differences in plant preferences between butterflies and syrphids (PERMANOVA, *F* = 2.22, *p* < 0.05, Appendix A4).

## 5 Discussion

Our study shows that newly established grasslands can effectively support diverse plant‐pollinator networks compared to old grasslands as they were sown with a highly diverse pollinator friendly seed mixture. Despite similar plant species richness to old grasslands, differences in plant assemblages and abundance of certain key plant species likely drove higher visitation and diversity in newly established grassland. Network structure and diet niches differed among pollinator groups, underscoring their distinct contributions to plant–pollinator networks.

### 5.1 Visitation Frequency and Diversity

The higher pollinator visitation frequency and diversity per plant species observed in newly established grasslands compared to old grasslands suggests that newly established grasslands are effectively fulfilling their role as pollinator‐supporting habitats within agricultural landscapes. Old grasslands and newly established grasslands exhibited similar plant species richness and flower abundance, but their plant assemblages differed significantly (Peer et al. 2024). This suggests that the plant assemblages—and likely higher abundance of key plant species, such as *Centaurea jacea* , *Knautia arvensis* , or *Onobrychis viciifolia—in* newly established grasslands offered more attractive floral resources, especially for solitary bees and syrphids (Warzecha et al. 2018). The fact that plant species identity explained much of the variance in the models—while site differences were negligible—emphasizes that the specific traits of individual plant species (such as flower morphology or nectar reward) are key drivers of pollinator attraction (Fornoff et al. 2017). However, another factor that may have influenced visitation rates is plant abundance. Previous studies have shown that plants arranged in packets receive more pollinator visits than solitary standing plants with single flowers (Akter et al. 2017; Herrera 2020). This could explain why, for example, *Galium mollugo* and *Knautia arvensis* were more frequently visited in newly established grassland than old grassland, as both plants were more abundant in newly established grassland. Additionally, a concentration effect may have played a role: newly established grasslands were mainly surrounded by arable land with few alternative floral resources, whereas old grasslands were often bordering on woody structures and other semi‐natural elements, potentially leading to more dispersed pollinator activity (Kleijn et al. 2011).

Previous studies in the Czech Republic and France found that plant‐pollinator networks were less diverse in newly established grassland due to lower plant species richness than in permanent semi‐natural grassland (Hadrava et al. 2022; Michelot‐Antalik et al. 2021). Our study shows that newly established grasslands with high plant diversity and a certain plant assemblage are indeed visited by a more diverse pollinator community. Since promoting pollinators is one of the main goals of newly established grasslands in an agricultural landscape, and encouraging plant‐pollinator communities with diverse interactions is suggested as an important conservation objective (Borchardt et al. 2021), our results are promising: they confirm that if a local and diverse seed mixture including pollinator friendly plants is used for grassland strips, a large number and variety of pollinators can be attracted.

### 5.2 Plant‐Pollinator Network Characteristics

While overall nestedness did not differ significantly between grassland types, we observed slightly higher nestedness in newly established grasslands compared to old grasslands for butterfly and syrphid networks: newly established grasslands contained a higher abundance of nectar‐rich plant species like *Knautia arvensis* , *Daucus carota* and *Centaurea jacea* , which were frequently visited by a diverse set of pollinator species: *Centaurea jacea*, for example, was frequently visited by butterfly generalists like *Coenonympha glycerion* and *Maniola jurtina*, which also visited a variety of other plant species. However, it was also predominantly visited by *Melanargia galathea*, a butterfly species with a clear preference for *Centaurea jacea* and a more specialized relationship with this plant (Kuster and Wirz 2002). This interaction structure illustrates the nestedness of the mutualistic network in newly established grasslands. Nestedness is considered an indicator of network stability, as it allows specialists to benefit from the population stability of generalist partners, thereby reducing the risk of secondary extinctions (Bascompte et al. 2003; Rohr et al. 2014). Our findings thus suggest that newly established grasslands supported potentially more stable networks, particularly those of butterflies and syrphids. According to Memtsas et al. (2022), nested structures also maintain plant diversity and support productivity through pollinators' contribution to successful plant reproduction and seed setting. However, it should be noted that the ecological implications of nestedness are not yet fully understood: some studies argue that specialized consumers could likely suffer from interspecific competition for limited resources and that increased niche overlap (nestedness) could therefore destabilize pollination networks (Blüthgen and Staab 2024).

Another essential component in maintaining the robustness and function of plant‐pollinator networks, especially under environmental change, is the presence of generalists (Resasco et al. 2021). In our study, generalist pollinators such as *Bombus pascuorum* , *Maniola jurtina*, and *Sphaerophoria scripta* were visiting many plant species across both grassland types. Likewise, *Centaurea jacea* and *Trifolium pratense* played key roles as highly visited plants, which is in line with previous studies on semi‐natural grassland (Michelot‐Antalik et al. 2021).

### 5.3 Diet Niches

Comparing pollinator groups, solitary bees had the highest network‐wide specialization, especially in old grasslands, which is consistent with conclusions drawn in previous research (Bosch et al. 2009; Raymond Heithaus 1979). At the same time, the NMDS analysis revealed that solitary bee species occupied distinct positions in the ordination space, indicating that while individual species were often specialized, they differ in their plant preferences. This high diversity within the group of solitary bees might be driven by morphological differences, like, for example, proboscis length (Cariveau et al. 2018). The whole group of solitary bees, however, was characterized by a preference for *Knautia arvensis* (Caprifoliaceae) in our study. *Knautia arvensis* is known to be a phenotypically generalized plant that attracts a wide range of pollinator species (Ebeling et al. 2008; Michelot‐Antalik et al. 2021). In our study, solitary bees accounted for a large proportion of the pollinator visits to *Knautia arvensis* , which is in contrast to previous work, which found butterflies, bumble bees, and syrphids to be most frequent visitors of *Knautia arvensis* (Ollerton et al. 2024). In contrast to solitary bees, bumble bee networks showed low specialization and high connectance. Nonetheless, compared to the other pollinator groups, they showed a certain preference for Fabaceae. This is not surprising as Fabaceae appear to be the major pollen and nectar source for most bumble bee species (Goulson et al. 2005; Wood et al. 2021). Results on plant family preferences in general showed a very high level of agreement with previous studies, for example, with plant pollinator networks in Ireland (Russo et al. 2022).

Syrphids are typically polylectic, visiting a broad variety of plants that offer abundant Nectar and Pollen (Branquart and Hemptinne 2000). However, level of specialization varies among syrphid subfamilies: in a previous study Eristalinae showed a strong preference for white flowers and Pipizinae visited mostly white and yellow flowers (Klecka et al. 2018). In this study we also recorded a preference of syrphids for white flowers, which is also reflected in the large numbers of syrphids in newly established grasslands visiting *Daucus carota* and *Galium mollugo* . *Galium* species were generally favored by syrphids—which is also reflected in their preference for the plant family of Rubiaceae. Syrphids' additional preference of Apiaceae in our study may be explained by the dependence of zoophagous syrphids on floral structures that are accessible to their short proboscis (Kormann 1975; van Rijn and Wäckers 2016). Butterflies—which showed the greatest difference to syrphid diet niches—showed a preference for purple flowers and plant families Fabaceae and Caryophyllaceae, which is in agreement with other research (Kolkman et al. 2022; Yurtsever et al. 2010). This preference is highly likely influenced by the morphological characteristics of butterfly mouthparts, particularly proboscis length, which enables access to nectar in deeper flowers, as is the case for many plant species from families Fabaceae and Caryophyllaceae (Szigeti et al. 2020).

The observed patterns of resource partitioning among pollinator groups highlight the value of diverse floral traits within grassland seed mixtures—offering complementary resources for a wider array of pollinators, which we suggest was one of the success factors for pollinator promotion in newly established grasslands.

### 5.4 Limitations

We want to point out that network indices such as connectance and network specialization can be influenced by sampling intensity (Dormann 2024). We minimized this risk by checking rarefied estimates, which confirmed our results. Additionally, our sampling was conducted from May to June and therefore does not account for interactions involving early‐flowering species in March and April. As plant–pollinator interactions are not static and may shift due to factors such as competitive pressure and floral resource availability (CaraDonna et al. 2017), studies with longer‐term data would help in assessing plant‐pollinator networks in newly established grassland.

## 6 Conclusions

Our study shows that newly established flower strips, when planted with diverse and certain key plant species (e.g., *Knautia arvensis* , *Daucus carota* , *Trifolium pratense* , *Centaurea jacea* ), can support diverse and potentially stable plant–pollinator networks comparable to or even exceeding those in old, permanent grasslands. However, key plant species certainly differ across biogeographic regions and should be assessed prior to establishment of new grasslands.

## Author Contributions

**Maria Peer:** data curation (lead), formal analysis (lead), investigation (equal), methodology (equal), visualization (lead), writing – original draft (lead), writing – review and editing (lead). **Sophie Kratschmer:** methodology (equal), supervision (supporting), writing – review and editing (supporting). **Raja Imran Hussain:** supervision (equal), writing – review and editing (supporting). **Aron Vogel:** investigation (equal). **Matthias Heer:** investigation (equal). **Simon Zwatz:** investigation (equal). **Dietmar Moser:** conceptualization (equal), funding acquisition (equal), methodology (equal), project administration (equal), supervision (equal), writing – review and editing (equal). **Thomas Frank:** conceptualization (equal), funding acquisition (equal), methodology (equal), project administration (equal), supervision (lead), writing – review and editing (equal).

## Funding

REGRASS 2 (2021–2023) was financed within the framework of the departmental research program via dafne.at with funds from the Federal Ministry of Agriculture, Forestry, Regions and Water Management. The BML supports applied, problem‐oriented, and practice‐oriented research (Project ID 101565).

## Conflicts of Interest

The authors declare no conflicts of interest.

## Supporting information


**Appendix S1:** ece373023‐sup‐0001‐AppendixS1.docx.

## Data Availability

All data and code supporting the findings of this study are available in the Zenodo repository at: https://doi.org/10.5281/zenodo.15805047.
