# Supplementary material for: Plant‐Pollinator Interactions in Grasslands Established on Arable Land
Source: Ecol Evol. 2026 Feb 2;16(2):e73023. doi: 10.1002/ece3.73023 (PMC12862277; doi:10.1002/ece3.73023)
Supplement: Supplementary file 1 — Appendix S1: ece373023‐sup‐0001‐AppendixS1.docx. [file ECE3-16-e73023-s001.docx]

**A1: Overview and location of study sites**

**
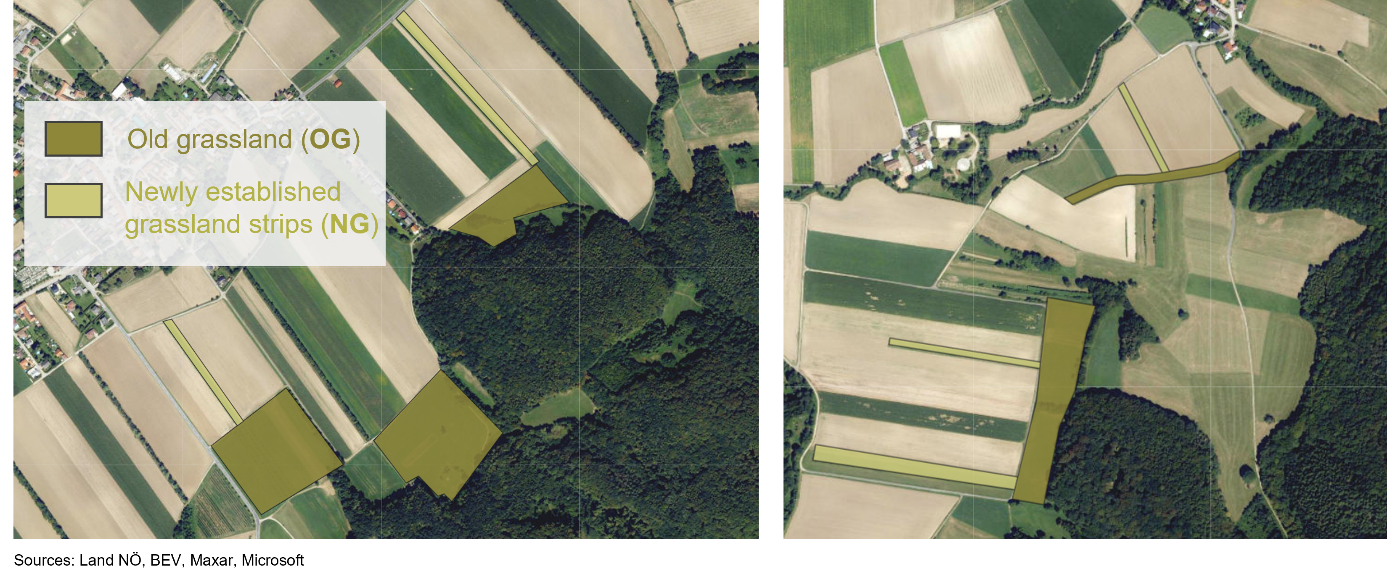
**

**Figure 1: Overview study sites:** Overview of the study site locations near the towns of Ollern (left) and Elsbach (right) in Lower Austria. The study area covered a radius of 1.75 km.

**A2: Visitation frequency**

Table 1: Generalized linear mixed-effects model (GLMM) results for visitation frequency per plant species of all pollinator groups.

|  | **Individuals (all groups)** | | | |
| --- | --- | --- | --- | --- |
| *Predictors* | *Incidence Rate Ratios* | *CI* | *Statistic* | *p* |
| (Intercept) | 0.21 | 0.12 – 0.39 | -5.07 | **<0.001** |
| Type [OG] | 0.71 | 0.50 – 0.98 | -2.16 | **0.031** |
| **Random Effects** | | | | |
| σ^2^ | 1.89 | | | |
| τ_00_ _Plant_ | 4.8 | | | |
| τ_00_ _Transect_ | 0.05 | | | |
| ICC | 0.72 | | | |
| N _Transect_ | 10 | | | |
| N _Plant_ | 135 | | | |
| Observations | 560 | | | |
| Marginal R^2^ / Conditional R^2^ | 0.004 / 0.721 | | | |

Table 2: Generalized linear mixed-effects model (GLMM) results for visitation frequency per plant species of solitary bees.

|  | **Individuals (solitary bees)** | | | |
| --- | --- | --- | --- | --- |
| *Predictors* | *Incidence Rate Ratios* | *CI* | *Statistic* | *p* |
| (Intercept) | 0.01 | 0.00 – 0.08 | -3.79 | **<0.001** |
| Type [OG] | 0.48 | 0.28 – 0.84 | -2.61 | **0.009** |
| **Random Effects** | | | | |
| σ^2^ | 6.62 | | | |
| τ_00_ _Transect_ | 0.00 | | | |
| τ_00_ _Plant_ | 15.79 | | | |
| N _Transect_ | 10 | | | |
| N _Plant_ | 118 | | | |
| Observations | 510 | | | |
| Marginal R^2^ / Conditional R^2^ | 0.020 / NA | | | |

Table 3: Generalized linear mixed-effects model (GLMM) results for visitation frequency per plant species of bumblebees.

|  | **Individuals (bumble bees)** | | | |
| --- | --- | --- | --- | --- |
| *Predictors* | *Incidence Rate Ratios* | *CI* | *Statistic* | *p* |
| (Intercept) | 0.00 | 0.00 – 0.06 | -4.06 | **<0.001** |
| Type [OG] | 1.00 | 0.45 – 2.21 | 0.00 | 0.997 |
| **Random Effects** | | | | |
| σ^2^ | 7.01 | | | |
| τ_00_ _Transect_ | 0.20 | | | |
| τ_00_ _Plant_ | 18.77 | | | |
| ICC | 0.73 | | | |
| N _Transect_ | 10 | | | |
| N _Plant_ | 114 | | | |
| Observations | 505 | | | |
| Marginal R^2^ / Conditional R^2^ | 0.000 / 0.730 | | | |

Table 4: Generalized linear mixed-effects model (GLMM) results for visitation frequency per plant species of syrphids.

|  | **Individuals (syrphids)** | | | |
| --- | --- | --- | --- | --- |
| *Predictors* | *Incidence Rate Ratios* | *CI* | *Statistic* | *p* |
| (Intercept) | 0.07 | 0.03 – 0.19 | -5.35 | **<0.001** |
| Type [OG] | 0.53 | 0.29 – 0.97 | -2.05 | **0.040** |
| **Random Effects** | | | | |
| σ^2^ | 3.05 | | | |
| τ_00_ _Plant_ | 5.52 | | | |
| τ_00_ _Transect_ | 0.19 | | | |
| ICC | 0.65 | | | |
| N _Transect_ | 10 | | | |
| N _Plant_ | 119 | | | |
| Observations | 515 | | | |
| Marginal R^2^ / Conditional R^2^ | 0.016 / 0.658 | | | |

Table 5: Generalized linear mixed-effects model (GLMM) results for visitation frequency per plant species of butterflies.

|  | **Individuals (butterflies)** | | | |
| --- | --- | --- | --- | --- |
| *Predictors* | *Incidence Rate Ratios* | *CI* | *Statistic* | *p* |
| (Intercept) | 0.01 | 0.00 – 0.06 | -5.04 | **<0.001** |
| Type [OG] | 0.83 | 0.55 – 1.27 | -0.84 | 0.400 |
| **Random Effects** | | | | |
| σ^2^ | 4.64 | | | |
| τ_00_ _Plant_ | 12.96 | | | |
| τ_00_ _Transect_ | 0.09 | | | |
| ICC | 0.74 | | | |
| N _Transect_ | 10 | | | |
| N _Plant_ | 126 | | | |
| Observations | 536 | | | |
| Marginal R^2^ / Conditional R^2^ | 0.000 / 0.738 | | | |

**A3: Visitation diversity**

Table 1: Generalized linear mixed-effects model (GLMM) results for visitation diversity per plant species of all pollinator groups.

|  | **Diversity (all groups)** | | | |
| --- | --- | --- | --- | --- |
| *Predictors* | *Incidence Rate Ratios* | *CI* | *Statistic* | *p* |
| (Intercept) | 0.22 | 0.13 – 0.37 | -5.84 | **<0.001** |
| Type [OG] | 0.75 | 0.58 – 0.96 | -2.24 | **0.025** |
| **Random Effects** | | | | |
| σ^2^ | 1.84 | | | |
| τ_00_ _Plant_ | 3.36 | | | |
| τ_00_ _Transect_ | 0.02 | | | |
| ICC | 0.65 | | | |
| N _Transect_ | 10 | | | |
| N _Plant_ | 135 | | | |
| Observations | 560 | | | |
| Marginal R^2^ / Conditional R^2^ | 0.004 / 0.65 | | | |

Table 2: Generalized linear mixed-effects model (GLMM) results for visitation diversity per plant species of solitary bees.

|  | **Diversity (solitary bees)** | | | |
| --- | --- | --- | --- | --- |
| *Predictors* | *Incidence Rate Ratios* | *CI* | *Statistic* | *p* |
| (Intercept) | 0.00 | 0.00 – 0.07 | -3.93 | **<0.001** |
| Type [OG] | 0.50 | 0.29 – 0.87 | -2.47 | **0.014** |
| **Random Effects** | | | | |
| σ^2^ | 6.53 | | | |
| τ_00_ _Transect_ | 0.00 | | | |
| τ_00_ _Plant_ | 15.98 | | | |
| τ_00_ _id_ | 0.00 | | | |
| N _Transect_ | 10 | | | |
| N _Plant_ | 118 | | | |
| N _id_ | 510 | | | |
| Observations | 510 | | | |
| Marginal R^2^ / Conditional R^2^ | 0.018 / NA | | | |

Table 3: Generalized linear mixed-effects model (GLMM) results for visitation diversity per plant species of bumble bees.

|  | **Diversity (bumble bees)** | | | |
| --- | --- | --- | --- | --- |
| *Predictors* | *Incidence Rate Ratios* | *CI* | *Statistic* | *p* |
| (Intercept) | 0.00 | 0.00 – 0.03 | -4.77 | **<0.001** |
| Type [OG] | 0.95 | 0.49 – 1.87 | -0.14 | 0.887 |
| **Random Effects** | | | | |
| σ^2^ | 6.47 | | | |
| τ_00_ _Transect_ | 0.12 | | | |
| τ_00_ _Plant_ | 18.67 | | | |
| τ_00_ _id_ | 0.09 | | | |
| ICC | 0.74 | | | |
| N _Transect_ | 10 | | | |
| N _Plant_ | 114 | | | |
| N _id_ | 505 | | | |
| Observations | 505 | | | |
| Marginal R^2^ / Conditional R^2^ | 0.000 / 0.744 | | | |

Table 4: Generalized linear mixed-effects model (GLMM) results for visitation diversity per plant species of syrphids.

|  | **Diversity (syrphids)** | | | |
| --- | --- | --- | --- | --- |
| *Predictors* | *Incidence Rate Ratios* | *CI* | *Statistic* | *p* |
| (Intercept) | 0.09 | 0.04 – 0.20 | -5.94 | **<0.001** |
| Type [OG] | 0.54 | 0.33 – 0.90 | -2.38 | **0.018** |
| **Random Effects** | | | | |
| σ^2^ | 2.77 | | | |
| τ_00_ _Plant_ | 3.75 | | | |
| τ_00_ _Transect_ | 0.10 | | | |
| ICC | 0.58 | | | |
| N _Transect_ | 10 | | | |
| N _Plant_ | 119 | | | |
| Observations | 516 | | | |
| Marginal R^2^ / Conditional R^2^ | 0.014 / 0.588 | | | |

Table 5: Generalized linear mixed-effects model (GLMM) results for visitation diversity per plant species of butterflies.

|  | **Diversity (butterflies)** | | | |
| --- | --- | --- | --- | --- |
| *Predictors* | *Incidence Rate Ratios* | *CI* | *Statistic* | *p* |
| (Intercept) | 0.02 | 0.01 – 0.08 | -6.00 | **<0.001** |
| Type [OG] | 1.11 | 0.84 – 1.47 | 0.74 | 0.458 |
| **Random Effects** | | | | |
| σ^2^ | 3.78 | | | |
| τ_00_ _Plant_ | 7.41 | | | |
| τ_00_ _Transect_ | 0.00 | | | |
| N _Transect_ | 10 | | | |
| N _Plant_ | 126 | | | |
| Observations | 536 | | | |
| Marginal R^2^ / Conditional R^2^ | 0.001 / NA | | | |

**A4: Diet niche overlap**

Table 1: Results of Adonis for all pollinator groups and pairwise adonis comparison (PERMANOVA).

|  | **PERMANOVA** | | | | |
| --- | --- | --- | --- | --- | --- |
| *Predictors* | *Sum of Squares* | *R2* | | *F* | *p* |
| Pollinator Group | 1.966 | 0.0432 | | 1.5349 | **0.015** |
| Residual | 43.549 | 0.9568 | |  |  |
| Total | 45.515 | 1 | |  |  |
| **Pollinator group pairs** |  | | | | |
|  | *F* | *R2* | *p* | | *p adjusted* |
| Bumble bees vs. butterflies | 1.496 | 0.037 | 0.089 | | 0.534 |
| Bumble bees vs. syrphids | 0.847 | 0.059 | 0.026 | | 0.156 |
| Bumble bees vs. wild bees | 0.625 | 0.028 | 0.116 | | 0.696 |
| Butterflies vs. syrphids | 2.22 | 0.041 | 0.002 | | **0.012** |
| Butterflies vs. wild bees | 1.328 | 0.019 | 0.144 | | 0.846 |
| Syrphids vs. wild bees | 1.026 | 0.016 | 0.398 | | 1 |

**A5: Rarefied estimates (Robustness Check for different sampling effort)**


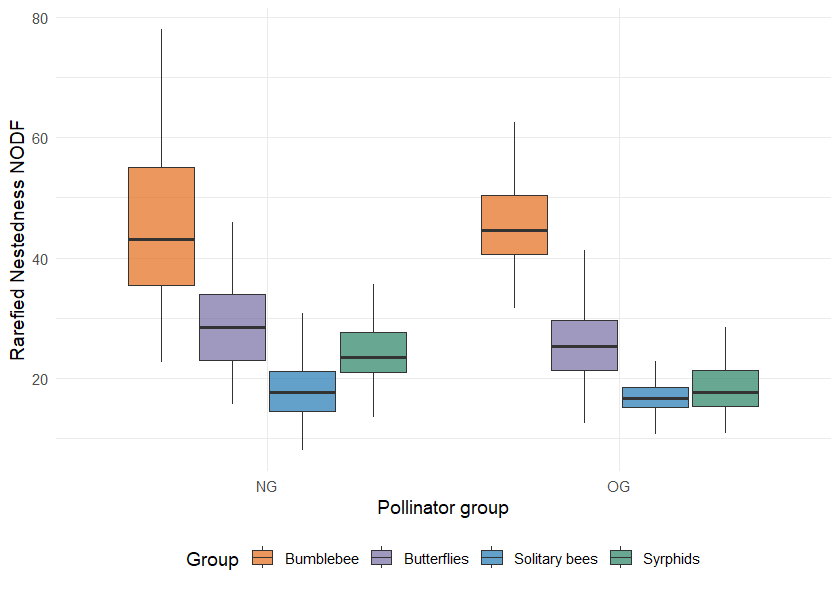

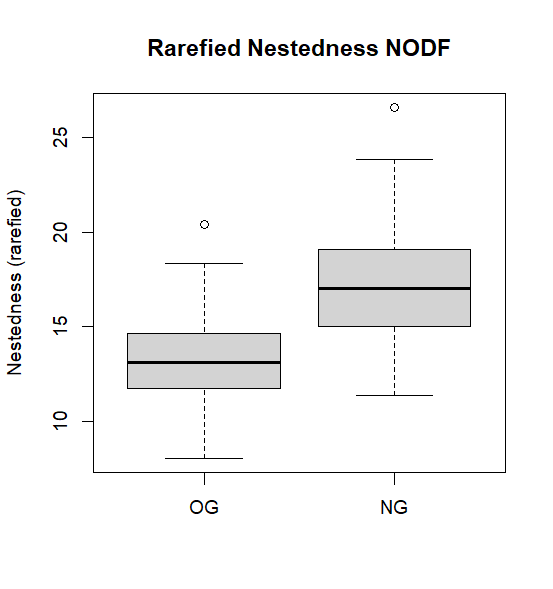

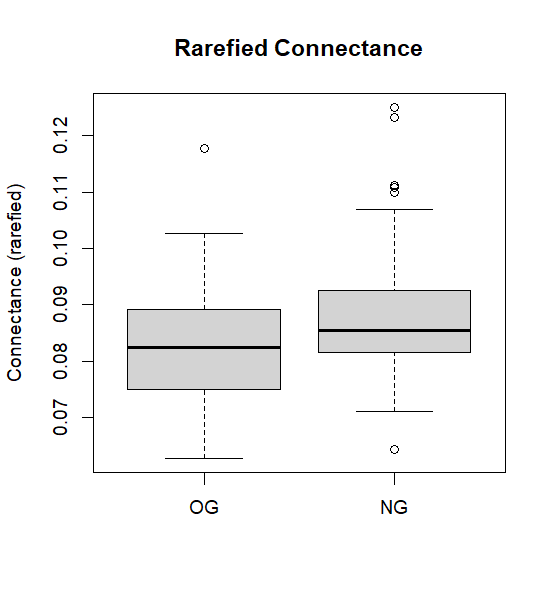

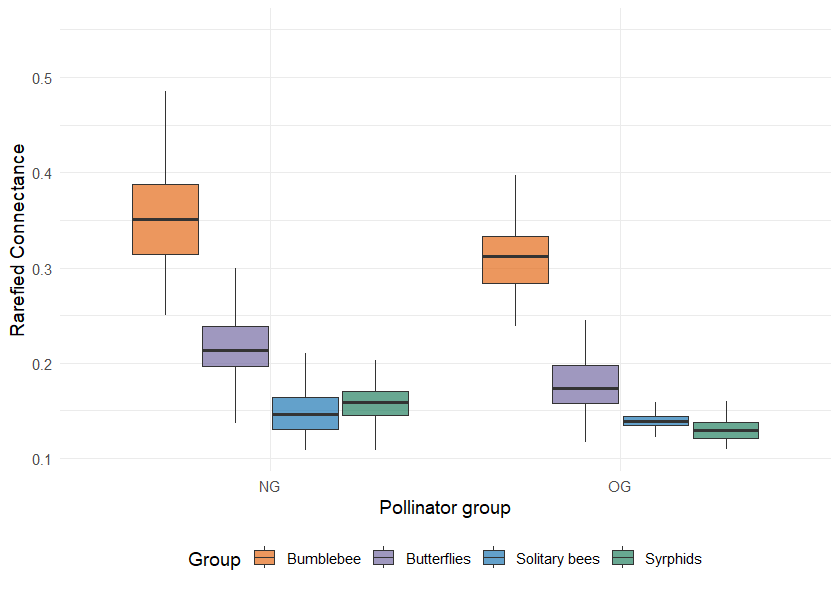

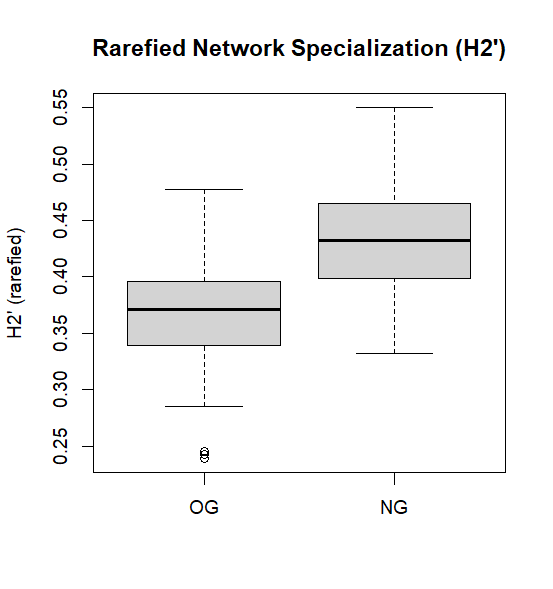

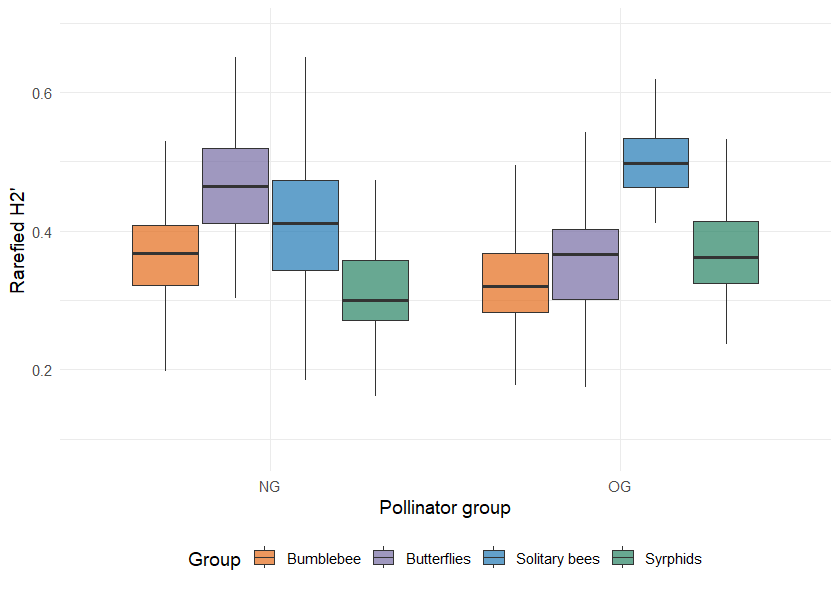


**A6: Mean coverage of plant species (based on Braun-Blanquet) with recorded pollinator interactions**

| **Speciesname** | **Mean coverage in NG** | **Mean coverage in OG** |
| --- | --- | --- |
| *Achillea millefolium* | 4.04 | 0.9 |
| *Agrostis gigantea* | 0.06 | 0 |
| *Ajuga reptans* | 0 | 0.4 |
| *Alopecurus pratensis* | 1.18 | 1.46 |
| *Anthoxanthum odoratum* | 2.58 | 2.58 |
| *Anthyllis vulneraria* | 1.46 | 0 |
| *Arrhenatherum elatius* | 17.28 | 10 |
| *Avenula pubescens* | 0 | 7.06 |
| *Briza media* | 1.1 | 0.58 |
| *Bromus erectus* | 3.62 | 11.66 |
| *Bromus hordeaceus* | 1.6 | 0.02 |
| *Bromus sterilis* | 0.18 | 0.06 |
| *Campanula patula* | 0 | 0.92 |
| *Cardamine matthioli* | 0 | 0.24 |
| *Carex caryophyllea* | 0 | 0.4 |
| *Carex flacca* | 0 | 0.02 |
| *Carex hirta* | 0 | 0.22 |
| *Carex pallescens* | 0 | 0.2 |
| *Centaurea jacea* | 11.08 | 1.52 |
| *Centaurea stoebe* | 0.28 | 0 |
| *Cerastium holosteoides* | 0.34 | 0.98 |
| *Cirsium arvense* | 0.2 | 0.08 |
| *Colchicum autumnale* | 0 | 2.68 |
| *Convolvulus arvensis* | 0.1 | 0 |
| *Crepis biennis* | 1 | 0.86 |
| *Cruciata laevipes* | 0 | 0.24 |
| *Cynosurus cristatus* | 0 | 0.06 |
| *Dactylis glomerata* | 1.94 | 4 |
| *Daucus carota* | 1.56 | 0.12 |
| *Dianthus carthusianorum* | 1.76 | 0.06 |
| *Equisetum arvense* | 0.5 | 0.06 |
| *Euphorbia verrucosa* | 0 | 0.02 |
| *Fallopia convolvulus* | 0.02 | 0.1 |
| *Festuca arundinacea* | 0 | 0.3 |
| *Festuca pratensis* | 8.9 | 7.38 |
| *Festuca rubra* | 0.96 | 0.52 |
| *Festuca rupicola* | 5.42 | 1.92 |
| *Festuca valesiaca* | 1.54 | 0 |
| *Filago vulgaris* | 0 | 0.06 |
| *Filipendula vulgaris* | 0 | 1.02 |
| *Fragaria viridis* | 0 | 0.14 |
| *Galium boreale* | 0 | 0.02 |
| *Galium mollugo* | 6.58 | 2.08 |
| *Galium verum* | 1.46 | 0.44 |
| *Geranium dissectum* | 0.24 | 0 |
| *Geranium pyrenaicum* | 0 | 0.06 |
| *Geum urbanum* | 0 | 0.1 |
| *Helianthemum canum* | 0 | 0.06 |
| *Heracleum sphondylium* | 0 | 0.22 |
| *Holcus lanatus* | 19.86 | 4.78 |
| *Hypericum perforatum* | 0.12 | 0 |
| *Inula oculus-christi* | 0.24 | 0 |
| *Knautia arvensis* | 3.38 | 0.7 |
| *Knautia maxima* | 0 | 0.2 |
| *Koeleria pyramidata* | 0.32 | 0 |
| *Lactuca serriola* | 0.06 | 0 |
| *Lathyrus pannonicus* | 0 | 0.02 |
| *Lathyrus pratensis* | 0.28 | 1.82 |
| *Leontodon hispidus* | 0.74 | 0.88 |
| *Leucanthemum vulgare* | 6.06 | 0.48 |
| *Linum catharticum* | 0 | 0.02 |
| *Lolium perenne* | 1.92 | 0 |
| *Lotus corniculatus* | 2.76 | 1.04 |
| *Luzula campestris* | 0 | 0.88 |
| *Lychnis flos-cuculi* | 0 | 0.28 |
| *Medicago lupulina* | 3.62 | 0.24 |
| *Medicago sativa* | 0.06 | 0.06 |
| *Melica ciliata* | 0.26 | 0 |
| *Melilotus officinalis* | 0.3 | 0 |
| *Myosotis arvensis* | 0.08 | 0.3 |
| *Onobrychis viciifolia* | 4.94 | 0.06 |
| *Ononis spinosa* | 0 | 0.34 |
| *Orobanche gracilis* | 0.08 | 0 |
| *Phleum pratense* | 0.42 | 0 |
| *Pimpinella major* | 0.06 | 0.08 |
| *Plantago lanceolata* | 2.28 | 5.32 |
| *Plantago media* | 0.18 | 0.08 |
| *Poa annua* | 0 | 0.02 |
| *Poa nemoralis* | 0 | 0.06 |
| *Poa pratensis* | 6.78 | 3.8 |
| *Poa trivialis* | 4.38 | 2.12 |
| *Polygala amara* | 0 | 0.02 |
| *Potentilla alba* | 0 | 0.52 |
| *Potentilla reptans* | 0 | 0.02 |
| *Primula veris* | 0 | 0.1 |
| *Prunella vulgaris* | 0.02 | 0.08 |
| *Ranunculus acris* | 0 | 1.42 |
| *Ranunculus bulbosus* | 0.2 | 1.28 |
| *Rhinanthus minor* | 0 | 1.22 |
| *Rumex acetosa* | 0 | 0.5 |
| *Rumex obtusifolius* | 0.06 | 0 |
| *Salvia pratensis* | 1.06 | 0.94 |
| *Sanguisorba minor* | 1.04 | 0.08 |
| *Silene nutans* | 0.06 | 0 |
| *Silene vulgaris* | 0.98 | 0 |
| *Taraxacum officinale* | 1.02 | 0.3 |
| *Taraxacum officinale agg.* | 0.8 | 0.42 |
| *Tragopogon orientalis* | 0 | 0.92 |
| *Trifolium campestre* | 0.14 | 1.12 |
| *Trifolium dubium* | 0 | 0.06 |
| *Trifolium montanum* | 0 | 0.48 |
| *Trifolium pratense* | 10.3 | 5.72 |
| *Trifolium repens* | 7.64 | 3.28 |
| *Trisetum flavescens* | 7.14 | 5.22 |
| *Veronica arvensis* | 0.3 | 0.1 |
| *Veronica chamaedrys* | 0 | 1.2 |
| *Vicia cordata* | 0 | 0.12 |
| *Vicia cracca* | 0.06 | 0 |
| *Vicia hirsuta* | 0.1 | 1.08 |
| *Vicia sepium* | 0.16 | 1.27 |
| *Viola hirta* | 0 | 0.06 |
| *Vulpia myuros* | 0.1 | 0 |
